# Low and High Speed Orthogonal Cutting of AA6061-T6 under Dry and Flood-Coolant Modes: Tool Wear and Residual Stress Measurements and Predictions

**DOI:** 10.3390/ma14154293

**Published:** 2021-07-31

**Authors:** Mahshad Javidikia, Morteza Sadeghifar, Victor Songmene, Mohammad Jahazi

**Affiliations:** Department of Mechanical Engineering, École de Technologie Supérieure, Montréal, QC H3W 1L8, Canada or Javidikia.m@gmail.com (M.J.); Victor.Songmene@etsmtl.ca (V.S.); mohammad.jahazi@etsmtl.ca (M.J.)

**Keywords:** orthogonal cutting, tool wear, residual stress, finite element model, aluminum alloy 6061-T6

## Abstract

The present research work aimed to study the effects of cutting environments and conditions on tool wear and residual stresses induced by orthogonal cutting of AA6061-T6. Cutting environments included dry- and flood-coolant modes and cutting conditions consisted of cutting speed and feed rate. A 2D finite element (FE) model was developed to predict tool wear and residual stresses and was validated by experimental measurements including machining forces, tool wear, and residual stresses. This was obtained by exploring various magnitudes of the shear friction factor and heat transfer coefficient and choosing proper coefficients using the calibration of the predicted results with the measured ones. The experimental results showed that the effect of cutting environment including dry and flood-coolant modes was negligible on machining forces. The experimental investigation also demonstrated that increasing feed rate raised machining forces, tool wear and residual stresses in both cutting environments. Low Speed Cutting (LSC) led to the highest value of tool wear and High Speed Cutting (HSC) provided the lowest values of resultant machining forces and residual stresses in both modes. Flood-coolant mode reduced tool wear and slightly decreased tensile residual stresses in comparison with dry mode. As a result, low feed rate and high-speed cutting under flood-coolant mode were proposed in order to improve tool wear and residual stress in orthogonal cutting of AA6061-T6.

## 1. Introduction

Machining operations are commonly used in the aerospace industry to produce the desired shape of components as reported by Javidikia et al. [1] and Touazine et al. [2]. Dry machining is frequently carried out due to the environmental and health regulations and reduction in the machining costs, as mentioned by Krolczyk et al. [3]. However, it can produce high cutting temperatures that could alter the dimensions and properties of the machined part. According to Brundtland et al. [4] and Khanna et al. [5], sustainability refers to a capacity that caters to the present human needs without endangering future generations to meet their needs. Sustainability can be realized in machining processes using cutting fluids, as mentioned by Sankaranarayanan et al. [6]. Khanna et al. [5] and Szczotkarz et al. [7] reported that during metal cutting operations, the high level of heat generated at the tool–workpiece–chip interfaces leads to high tool wear and low surface integrity. To tackle this problem, Khanna et al. [5] and Adler et al. [8] stated that cutting fluids can be utilized to reduce the temperature in the cutting region and also perform functions such as lubrication and flushing of chips. The most widespread type of cutting fluids used in machining operations is water-based emulsions, leading to a popular cutting process called flood-coolant (wet) machining, as expressed by Szczotkarz et al. [7].

Orthogonal cutting of aluminum alloys is an important machining operation in the aerospace industry, particularly as initial step for further and more complex operations. According to Javidikia et al. [9], one of the major challenges in othrogonal cutting of aluminum alloys is the occurrence of non-uniform cutting temperatures and machining forces. A previous research study by Hu and Huang [10] on turing of AISI4340 steel demonstrated that machining forces and temperature are the fundamental factors that determine the extent of residual stresses and tool wear. High tool wear can result in reduction in tool life and decrease the quality of the components, thereby increasing machining costs. Large tensile residual stresses can significantly affect fatigue life and corrosion resistance of machined components, which lead to crack propagation, as mentioned by Javidikia et al. [1] and Sadeghifar et al. [11], who carried out research on surface integrity induced by turning of AA6061-T6 and residual stresses and machining characteristics generated by orthogonal turning of 300M Steel, respectively. Based on the research work by Javidikia et al. [9], the machining with the cutting speed below 900 $\left(m/\min\right)$ is considered as LSC, while above 900 $\left(m/\min\right)$ is known as HSC. Therefore, different experimental and numerical studies have been carried out to improve tool wear and residual stress induced by machining processes.

Leppert and Peng [12] carried out an experimental study to investigate the effects of cutting environments and conditions on residual stresses after turning AISI 316L steel. The results demonstrated that by properly selecting cutting parameters and residual stresses in dry mode could be smaller or comparable with those in wet mode. Cantero et al. [13] experimentally analyzed tool wear mechanisms in finishing turning of Inconel 718 with three carbide diamond-shaped cutting inserts under dry and wet cutting environments. They found that tool wear was higher in dry mode.

MacGinley and Monaghan [14] simulated tool wear, temperature and stress distributions in the workpiece in orthogonal turning Inconel 718 with uncoated and coated tools using the Forge software – Version 2 (produced by Transvalor). Good agreement was observed between the simulated results and the experimental ones. Yen et al. [15] implemented a tool wear model into FE modeling of orthogonal machining of AISI 1045 steel with uncoated carbide tools. They employed a special simulation module called “Konti-Cut” in order to simulate the cutting process for a sufficiently long cutting time using the DEFORM software – Version 11.0 (produced by Scientific Forming Technologies Corporation (SFTC)). The results showed that this approach tended to underestimate the wear rates and, consequently, some wear constants were required for the FE model to be calibrated accurately.

Xie et al. [16] carried out FE simulations of tool wear in orthogonal turning of AISI 1045 steel by integrating Abaqus/Explicit and Abaqus/Standard – Version 6.2 (produced by Dassault Systèmes). Significant discrepancy was observed between the experimental and predicted results for both flank wear and crater wear. This discrepancy was attributed to the difference of the characteristic equation of tool wear and the tool wear data available in the literature, the simplified friction model, the difference in the chemical composition and heat treatment of the workpiece used in the experiment and simulation, and the poor mesh control at the tool–chip interface. Coelho et al. [17] performed FE modeling of orthogonal turning of AISI 4340 steel using the Abaqus software to predict tool wear and machining forces with uncoated and coated carbide inserts. Good agreement was observed between predictions and measurements.

Soliman et al. [18] analyzed the effect of feed rate on tool wear in orthogonal cutting of A36 steel using uncoated carbide insert. The Abaqus software was used to develop a 2D finite element model validated by experimental investigation. The results showed that crater wear increased with increasing feed rate.

The influence of orthogonal cutting parameters on residual stresses was assessed. Jomaa et al. [19] studied the effects of cutting speed and feed rate on residual stresses in orthogonal cutting of AA7075-T651. They showed that the hoop surface residual stress was compressive in low cutting speed and the axial surface residual stress became tensile with increasing cutting speed. They also reported that the effect of cutting speed on residual stresses was higher when lower feed rates were employed. Outeiro et al. [20] analyzed residual stress variations using different cutting parameters in orthogonal turning of AISI 316L steel with uncoated and TiC/Al_2_O_3_/TiN-coated tungsten carbide tools. The results showed that the surface residual stresses remained almost constant and increased using uncoated and coated tools, respectively; in contrast, the surface residual stress rose with increasing feed rate.

Maranhao and Davim [21] developed a FE model of orthogonal cutting of AISI 316 steel to predict the effect of feed rate on residual stress using the AdvantEdge software (produced by Third Wave Systems). They concluded smaller feed rates caused the lower residual stresses. Mohammadpour et al. [22] examined the influence of cutting speed and feed rate on the distribution of residual stresses in orthogonal cutting of AISI 1045 steel using the SuperForm software – Version 2005 (produced by MSC.Software Corporation). The results displayed that raising cutting speed and feed rate increased residual stresses.

Moussa et al. [23] studied the effect of cutting speed and feed rate on residual stresses induced by the orthogonal cutting of AISI 316L steel. They found that the residual stress in the machined subsurface decreased when cutting speed rose and depth of cut decreased. Qi et al. [24] analyzed the effect of different machining parameters on surface residual stress during dry cutting AISI 1045 steel. Their results showed that the surface residual stress is not sensitive to the variation of cutting speed. Moreover, they found that residual stress considerably increased with increasing cutting depth.

Sadeghifar et al. [11] conducted FE modeling of cutting temperature, cutting and thrust forces, and residual stresses in dry orthogonal turning of 300M steel using the Abaqus software – Version 6.14. The results showed that higher cutting speed and lower feed rate were desirable to decrease residual stresses when machining forces, temperature, and material removal rate were constrained. Muñoz-Sanchez et al. [25] studied the impact of tool wear on residual stresses in machining of AISI 316L steel using the Abaqus/Explicit and Abaqus/Standard – Version 6.4-1. The results demonstrated that the residual stress increased when worn tools were employed compared to the fresh ones.

As seen in the above-mentioned papers, very little information is available in the published literature on the effect of different cutting environments and high-speed machining on tool wear induced by orthogonal cutting of metals. The information is even scarcer when it comes to different cutting environments such as dry and flood-coolant modes for orthogonal cutting of aluminum alloys.

In the present research work, the effects of cutting environments and conditions on machining forces, tool wear, and residual stresses induced by orthogonal cutting of AA6061-T6 were investigated. Special attention was devoted to examining the influence of low speed cutting and high speed cutting on tool wear and residual stress. The developed 2D FE model was experimentally validated using cutting forces, tool wear, and residual stress. The variations of machining forces, tool wear, and residual stress with cutting environments and conditions were analyzed and discussed.

## 2. Experimental Tests

**Figure 1:**
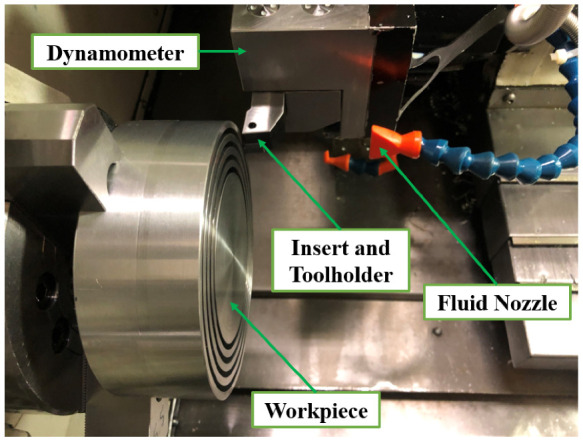
The experimental set-up of orthogonal machining.

Orthogonal cutting tests were conducted using a MAZAK-NEXUS 100-II M CNC machine (Florence, KY, USA). The workpiece was a 150-mm diameter and 120-mm-length cylinder made of AA6061-T6. The tool was made of uncoated carbide (ISO CCGX 120408-AL H10) and a right-hand tool holder of SCLCR 2020 K12 was used to hold the inserts. For each experimental test, a new insert was employed to provide similar conditions for all the tests. The samples were groove machined to form tube-shaped workpiece with a 4 mm thickness. The orthogonal cutting tests were performed for the cutting conditions listed in Table 1 with the tool geometry consisting of a edge radius of $r_{\beta }$=0.02 mm, a rake angle of $\gamma_{o}$= 17.5 degrees, and a clearance angle of $\alpha_{o}$ = 7 degrees under dry and flood-coolant modes. A Kistler (type 9121) three-component piezoelectric dynamometer (Winterthur, Switzerland) was utilized to measure machining forces. The acquisition of force signals was carried out with LabVIEW software and data treatment was conducted using MATLAB codes. The experimental set-up of the orthogonal cutting is shown in Figure 1. The utilized flood-coolant was OEMETA with the flow rate of 7200 mL/min.

**Figure 2:**
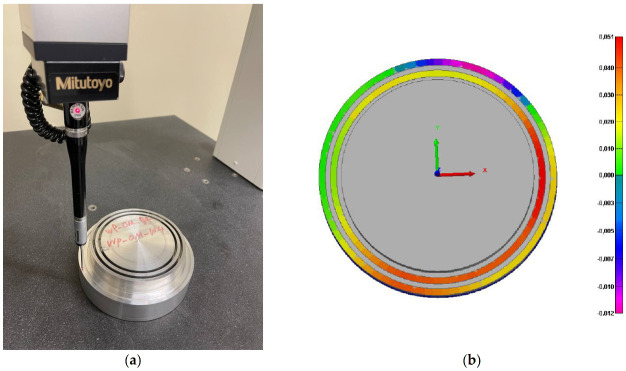
(**a**) Measurement of surface profile of the workpiece using a coordinate measuring machine and (**b**) the map of the surface profile.

**Figure 3:**
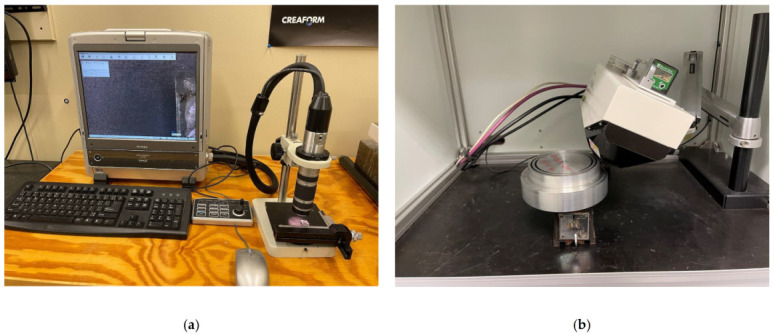
(**a**) Digital Microscope VHX-500F for measuring tool wear and (**b**) a Pulstec μ-X360n XRD machine for measuring residual stresses.

A Mitutuyo Crysta-Apex C Coordinate Measuring Machine (CMM) (Kanagawa, Japan) was used to evaluate the homogeneity of the final machined surface before conducting the residual stress measurements, as shown in Figure 2a. As displayed in Figure 2b, the final machined surface is homogenous all around the surface due to the negligible variation of the height from the reference surface. Digital Microscope KEYENCE VHX-500F (Osaka, Japan) was used to observe and measure the length of tool wear as portrayed in Figure 3a. A Pulstec µ-X360n X-Ray Diffraction machine (Nakagawa, Japan) was used to measure surface residual stresses as displayed in Figure 3b. This machine uses a Debye–Scherrer ring image based on a diffracted cone and cos α method to measure and calculate residual stresses. Moreover, the X-ray incidence angle and X-ray irradiation time were set as 25 degrees and 20 s, respectively. The Bragg’s angle and crystallographic plane were 139.3 degrees and {311}, respectively. It needs mentioning that residual stress was measured on all the machined samples at four points and was averaged.

## 3. Results and Discussion

### 3.1. Machining Force Analysis

**Figure 4:**
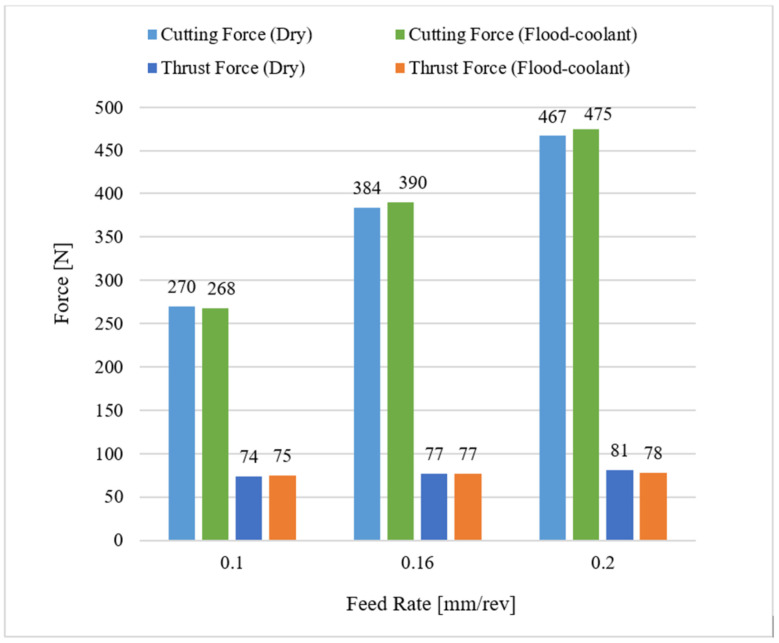
Variation of cutting and thrust forces with feed rate in dry and flood-coolant modes.

The effect of feed rate on machining forces consisting of cutting force ($F_{c}$) and thrust force ($F_{t}$) was studied under dry and flood-coolant modes for Test Nos. 3, 5, and 6, where the cutting speed was fixed at 950 m/min, as shown in Figure 4. As observed in this figure, the resultant machining forces increased with raising feed rate for both modes. This is because higher feed rates lead to larger tool–chip contact area and pressure, resulting in larger magnitudes of machining forces [9,11].

**Figure 5:**
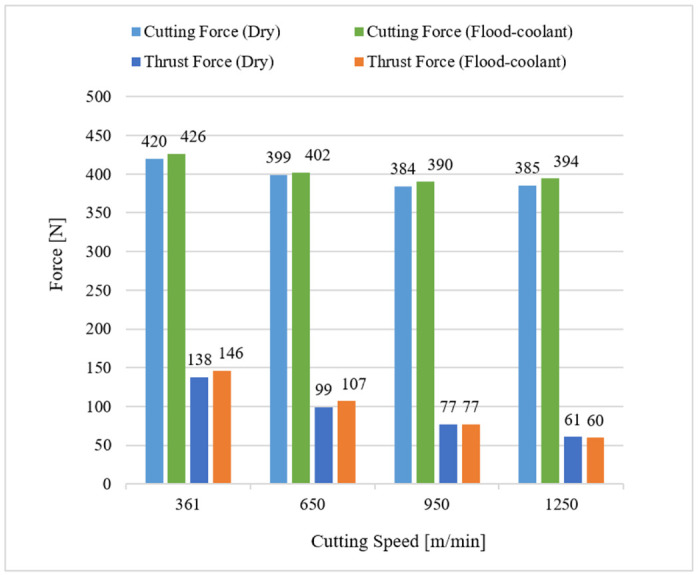
Variation of cutting and thrust forces with cutting speed in dry and flood-coolant modes.

Figure 5 displays the influence of cutting speed on $F_{c}$ and $F_{t}$ for both modes as detailed in Test Nos. 1, 2, 3, and 4 of Table 1. As presented in this figure, increasing cutting speed from LSC to HSC decreased the resultant machining forces under dry and flood-coolant modes. This can be attributed to the fact that a larger cutting speed produces larger frictional and plastic works, leading to more thermal softening of material, and consequently, generates lower forces during machining [9,11].

Furthermore, the results reported in Figure 4 and Figure 5 reveal that changing cutting environments from dry to flood-coolant was negligeable on the machining forces. This could be related to the fact that, in a cutting process, applying a coolant reduces the cutting temperature, resulting in less thermal softening of the machined material, which therefore increases the resultant machining forces. On the other hand, a coolant can also have a lubrication effect. Indeed, applying a coolant also decreases the generated friction between the tool and workpiece, and consequently reduces the resultant forces. Therefore, the net effect of these two phenomena does not change the resultant forces in flood-coolant mode compared with dry mode.

### 3.2. Tool Wear Analysis

**Figure 6:**
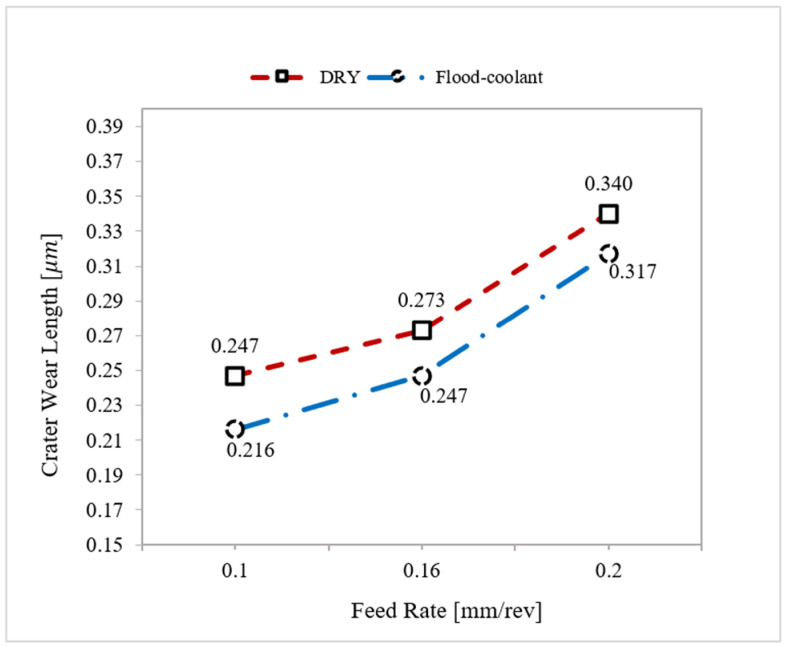
Variation of crater wear length with feed rate in dry and flood-coolant modes.

The results reported in Figure 6 based on Tests Nos. 3, 5, and 6, in which cutting speed was fixed at 950 m/min, cleary demonstrate that the length of crater wear increased with increasing feed rate. This is due to the fact that raising feed rate increases the tool-chip contact pressure, contact area and friction, resulting in higher crater wear [26].

**Figure 7:**
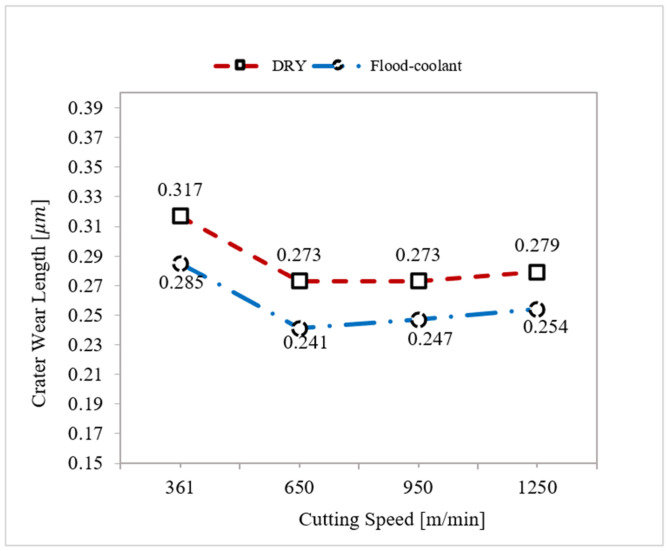
Variation of crater wear length with cutting speed in dry and flood-coolant modes.

The variation of crater wear with cutting speed was investigated under dry and flood-coolant modes for Test Nos. 1, 2, 3, and 4 for feed rate fixed at 0.16 mm/rev, as shown in Figure 7. According to this figure, the largest length of crater wear occurred in the cutting speed of 361 m/min (LSC) and it then dropped with increasing cutting speed from 361 m/min to 650 m/min. Moreover, as shown in this figure, the length of crater wear remained almost constant with increasing cutting speed from 650 m/min to 1150 m/min (HSC). The observed behavior is probabaly related to the presence of higher machining forces when the cutting speed of 361 m/min was used in the experiments, as reported in Figure 5, and, consequently, induced more pressure and friction on the cutting tool, leading to more crater wear.

**Figure 8:**
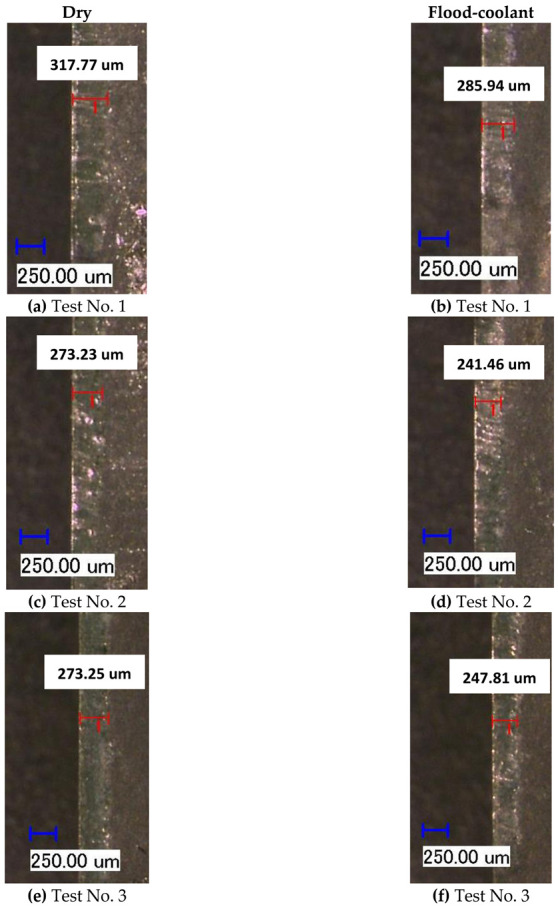

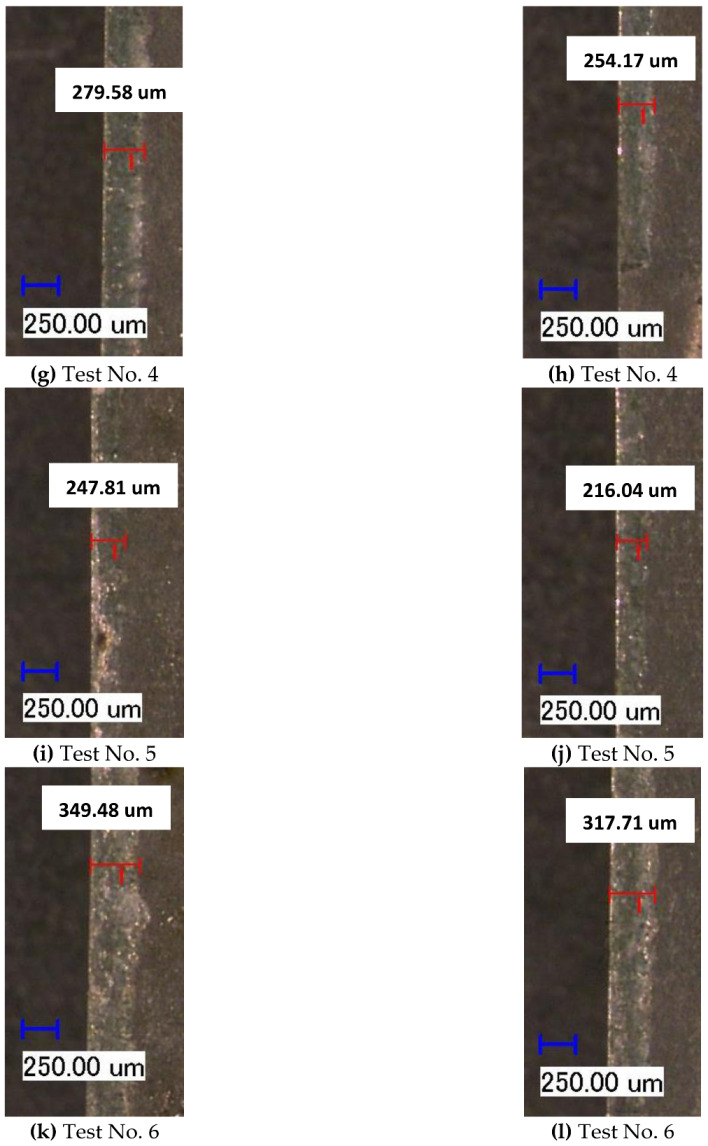
The length of crater wear in both dry and flood-coolant modes for all the six cutting conditions.

As seen in Figure 6 and Figure 7, flood-coolant mode improved the length of crater wear compared with dry mode. This result is in agreement with that obtained by Kishawy et al. [27], who reported reduced tool wear in flood-coolant high-speed milling of A356 aluminum alloy compared to the dry mode. This is attributed to the fact that applying flood-coolant led to lower friction and heat generation and, as a result, reducing the crater wear. It needs mentioning that there is a direct relationship between “the cutting temperature and friction” where tool wear increases with increasing temperature and friction [10]. The length of crater wear in both dry and flood-coolant modes for all the six cutting conditions are shown in Figure 8.

Accordingly, a combination of low feed rate and high cutting speed in flood-coolant mode is recommended to reduce the length of crater wear.

### 3.3. Residual Stress Analysis

**Figure 9:**
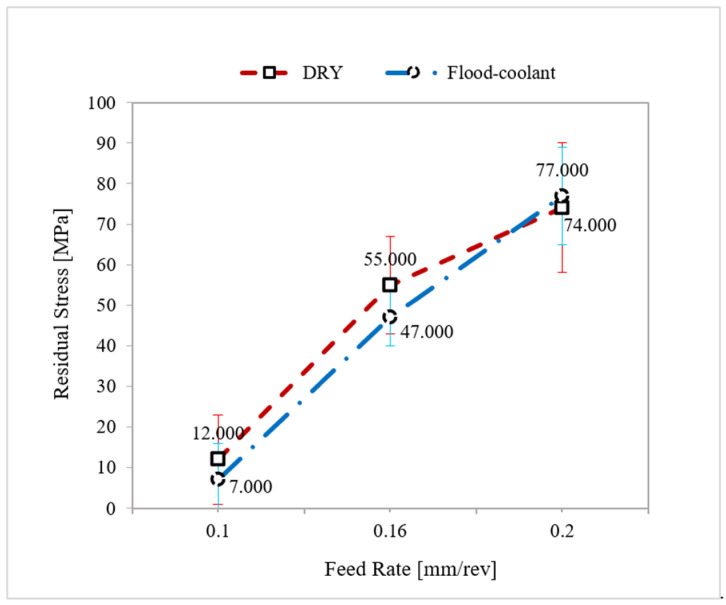
Variation of residual stresses with feed rate in dry and flood-coolant modes.

The plots of the variation of residual stresses with feed rate and the corresponding standard deviations in dry and flood-coolant modes for Test Nos. 3, 5, and 6 for cutting speed fixed at 950 m/min were displayed in Figure 9. As seen in this figure, residual stresses increased with raising feed rate for both modes. This is because increasing feed rate increases the tool–chip contact area and the frictional heat, which increases temperature and residual stresses [1,11].

**Figure 10:**
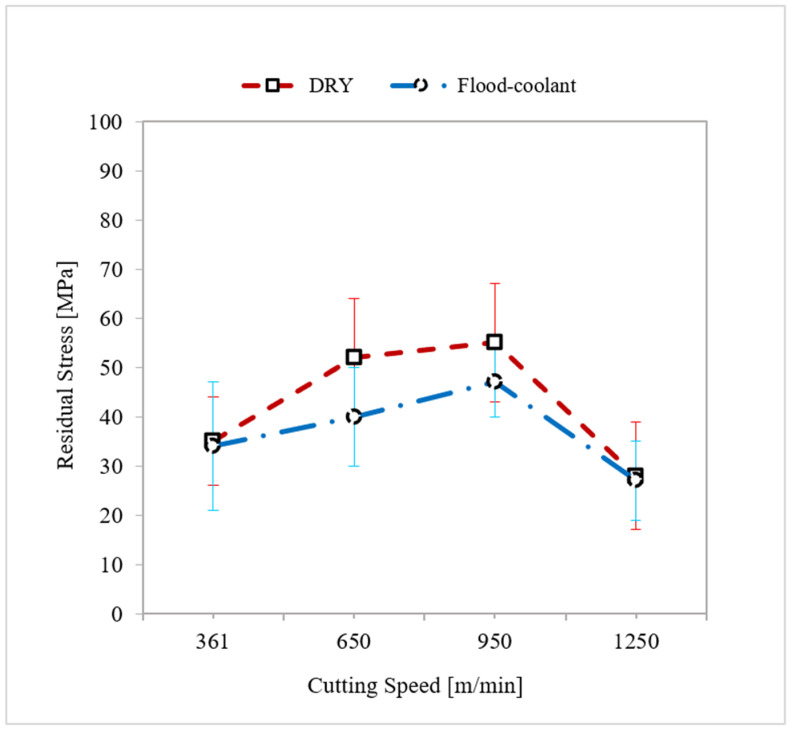
Variation of residual stresses with cutting speed in dry and flood-coolant modes.

A study of the impact of cutting speed on residual stresses and their corresponding standard deviations was carried out under dry and flood-coolant modes for Test Nos. 1, 2, 3, and 4, in which feed rate was kept fixed at 0.16 mm/rev, as shown in Figure 10. Based on this figure, residual stresses increased with cutting speed in the range of 361 m/min to 950 m/min and then dimineshed from the cutting speed of 950 m/min to 1150 m/min in both dry and flood-coolant modes. In a cutting process, an increment in cutting speed increases the plastic work, $\sigma_{fl}{\dot{\varepsilon }}_{p},$ and the frictional work, $\tau V_{Ch}$, [28], in which $\sigma_{fl}$, ${\dot{\varepsilon }}_{p}$, τ, and $V_{Ch}$ are flow stress, effective plastic strain rate, frictional shear stress at the tool–chip contact face, and chip velocity along the tool-chip interface, respectively. These works increase the generated heat and, consequently, raise the temperature and residual stresses [29,30]. In contrast, an increment in cutting speed raises material removal rate (MRR), which increases the heat evacuation and, as a result, reduces temperature [9] and residual stresses [11,29,30]. The competition between these two phenomena determines the nature (tensile or compressive) and amplitude of the residual stresses.

As shown in Figure 9 and Figure 10, in general, the residual stresses slightly decreased by applying coolant due to reduction in the cutting temperature and friction. Since the magnitude of the resultant machining forces remained almost constant for the two modes (Figure 4 and Figure 5), it can be concluded that the variation of the residual stresses in orthogonal cutting of AA6061-T6 was more dependent on thermal load than mechanical load.

As a result, a combination of low feed rate and high cutting speed in flood-coolant mode is proposed in order to obtain lower residual stresses. Although turning at high cutting speed led to almost similar residual stresses to turning at low cutting speed, the productivity is higher at HSC as a consequence of larger metal removal rates.

## 4. Finite Element Modeling

In this research study, Finite Element (FE) Method was used to simulate the orthogonal cutting of AA6061-T6. The DEFORM^TM^ software – Version 11.0 was employed to predict the responses. Accurate predictions of machining forces, tool wear, and residual stresses are crucial for selecting the optimal machining parameters to improve the tool performance and the surface integrity of components as the end goal of the machining industry. The mathematical formulation of the analysis is based on an updated Lagrangian formulation and implicit integration method for large plastic deformation analysis.

The equations of motion during the orthogonal cutting process at a specific instant of time are expressed as [11]:

(1) $$\left[M\right]\{\ddot{U}\}+\left\{R_{\mathrm{int}}\right\}=\left\{R_{ext}\right\}$$

where $\left[M\right]$ is the mass matrix, $\left\{\ddot{U}\right\}$ is the acceleration vector ($\left\{U\right\}$ is the displacement), and $\left\{R_{\mathrm{int}}\right\}$ and $\left\{R_{ext}\right\}$ are the vectors of internal and external forces, respectively. The effect of damping is ignored and, consequently, $\left\{R_{\mathrm{int}}\right\}$ is equal to

(2) $$\left\{R_{\mathrm{int}}\right\}=\left[C_{d}\right]\left\{\dot{U}\right\}+\left[K_{s}\right]\left\{U\right\}≅\left[K_{s}\right]\left\{U\right\}\;\mathrm{where}\;\left[C_{d}\right]≅0$$

where $\left[C_{d}\right]$ and $\left[K_{s}\right]$ are the damping and stiffness matrices, respectively. In addition, $\left\{R_{ext}\right\}$ is the external forces applied during cutting including the reaction forces at the supports.

Heat transfer occurring during the machining process is described as [9]:

(3) $$\left[C_{T}\right]\;\{\dot{T}\}+\left[K_{T}\right]\;\left\{T\right\}\;=\{{\dot{Q}}_{g}\}$$

in which $\left[C_{T}\right]$ and $\left[K_{T}\right]$ are the volumetric heat capacitance and thermal conduction matrices, respectively. Moreover, $\left\{{\dot{Q}}_{g}\right\}$ is the total heat generation in the machining process.

The thermal contact between the tool and workpiece is defined by considering heat conduction through the tool–chip contact face from the chip to the tool during the cutting process. The heat conduction is calculated as:

(4) $$Q=h_{\mathrm{int}}\;\left(T_{wp}-T_{t}\right)$$

where $h_{\mathrm{int}}$ is heat transfer coefficient, $T_{wp}$ and $T_{t}$ are the workpiece and tool’s temperature at the tool–chip interface. An initial temperature of 20 ℃ (room temperature) was considered to both tool and workpiece.

Convection heat transfer occurs between the workpiece and the environment according to the following formula [11]:

(5) $$Q=h\;\;\left(T_{wp}-T_{a}\right)$$

in which h is convection heat transfer coefficient, and $T_{wp}$. and $T_{a}$ are the workpiece and ambient (room) temperature. In the present work, $h_{\mathrm{int}}$ and h were calibrated by comparing the predicted results of machining forces, tool wear, and residual stress with the corresponding experimental ones.

The Johnson–Cook material constitutive model was utilized to model the plastic deformation of the workpiece material during the cutting process as:

(6) $$\sigma_{fl}=\left[A+B{\left(\varepsilon \right)}^{n}\right]\left[1+C\ln\left(\frac{\dot{\varepsilon }}{{\dot{\varepsilon }}_{0}}\right)\right]\left[1-{\left(\frac{T-T_{room}}{T_{melt}-T_{room}}\right)}^{m}\right]$$

where $\sigma_{fl}$ is the flow stress, ε. the plastic strain, $\dot{\varepsilon }$ the plastic strain rate $\left(s^{-1}\right)$, ${\dot{\varepsilon }}_{0}$ the reference plastic strain rate $\left(s^{-1}\right)$, T (Co) the workpiece temperature, Tmelt (Co) the melting temperature of the workpiece, and Troom(Co) the room temperature. Additionally, $A\;\left(\mathrm{MPa}\right)$ is the initial yield strength, $B\;\left(\mathrm{MPa}\right)$ the hardening modulus, C the strain rate sensitivity coefficient, n the hardening coefficient, and m the thermal softening coefficient. Table 2 presents the Johnson–Cook constants of AA6061-T6.

Previous research indicated that aluminum alloys tend to adhere to the tool at the tool–chip interface during cutting which creates a sticking zone [30]. The Coulomb and Zorev models cannot predict the frictional behavior accurately due to lack of relative sliding at the tool–chip interface. Hence, the shear friction model was utilized to model the mechanical contact between the tool and the workpiece as follows [9]:

(7) $$\tau =m_{f}\tau_{Chip}$$

where $m_{f}$ is the shear friction coefficient and $\tau_{Chip}$ is the shear flow stress in the chip at the tool–chip interface. In this study, the shear friction coefficient was calibrated by comparing the present simulated machining forces, tool wear, and residual stress with the experimentally measured results.

Several tool wear models were commonly used in FE simulation of metal cutting processes such as Taylor’s extended equation, Takeyama’s wear model, and Usui’s wear model [31]. The Usui’s wear model has been extensively used in machining simulations because of consistent experimental validations of its predictions for a wide range of machining processes and conditions [10,26,32,33,34,35,36]. Therefore, in the present research study, Usui’s wear model was used to simulate tool wear as follows:

(8) $$\frac{dw}{dt}=A\;\sigma_{t}\;V\exp\left(\frac{-B}{T}\right)$$

in which V is the sliding velocity, T interface temperature, $\sigma_{t}$ interface pressure, and w tool wear. In addition, the values of the Usui’s model constants (A and B) for uncoated carbide tools are given as [31,37,38]:

$A=7.8\times 10^{-9}$ and $B=5.302\times 10^{3}$ for T<1150 K

$A=1.198\times 10^{-2}$ and $B=2.195\times 10^{4}$ for T≥1150 K

According to a previous research study by the same authors [9], the maximum temperature of the tool induced by orthogonal cutting of AA6061-T6 using uncoated carbide inserts occurred at the tool rake face and their values were much less than 1150 K. Thus, in the present simulations, the Usui’s model constants were considered as $A=7.8\times 10^{-9}$ and $B=5.302\times 10^{3}$.

Using cutting fluids during cutting processes can bring along two main functions consisting of cooling and lubrication. These two main functions can affect the friction and heat convection and conduction during the cutting processes [39].

**Figure 11:**
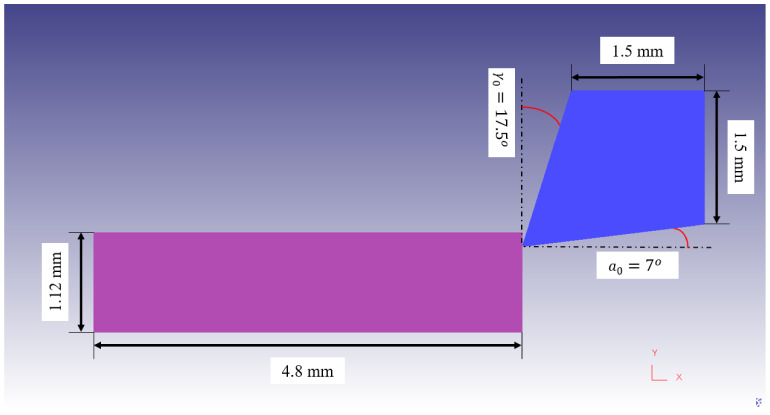
The geometry and dimensions of the tool and workpiece in FE modeling.

In the present simulations, a rectangular workpiece with dimensions of 4.8 mm × 1.12 mm was used with an elastic–plastic behavior. The workpiece was meshed with 3424 linear quadrilateral elements and 3540 nodes. The tool material was considered as a rigid body and was meshed with 1981 elements and 2080 nodes. Figure 11 exhibits the tool and workpiece’s geometries. Mesh windows were assigned to the workpiece and tool in order to have a high-quality fine mesh in the cutting zone. The workpiece and tool’s material properties are adopted from Ref. [9].

**Figure 12:**
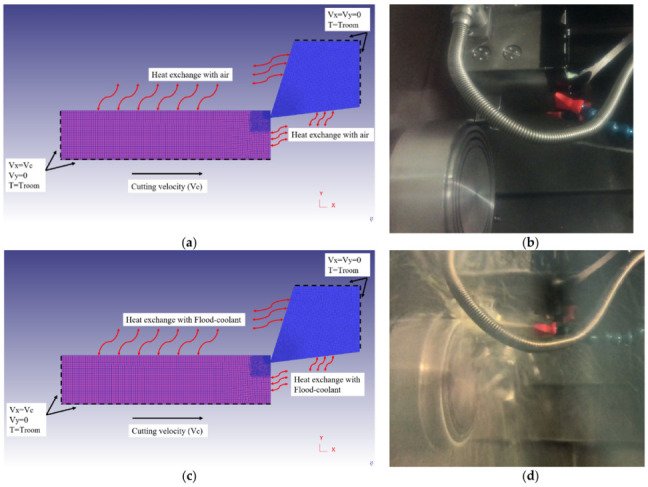
Thermal and mechanical boundary conditions in the cutting process of (**a**) dry-FE model, (**b**) dry-Exp. test, (**c**) flood coolant-FE model, (**d**) flood coolant-Exp. test.

**Figure 13:**
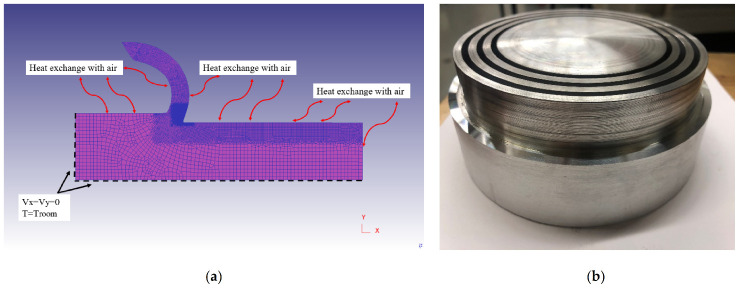
Thermal and mechanical boundary conditions in the stress relaxation process of (**a**) FE model and (**b**) Exp. test.

Residual stresses were modeled and extracted during two steps: cutting and stress relaxation processes. In the first step, cutting is conducted to reach the steady-state condition in which machining forces, temperature, strains and stresses, and chip thickness remain almost constant with time. As displayed in Figure 12, in both horizontal and vertical directions, the top and right sides of the cutting tool are fixed. Moreover, the bottom and left sides of the workpiece are fixed in vertical direction. The workpiece material moves through the fixed tool in the horizontal direction. The sides of the workpiece and tool which are far from the cutting zone and are retained at ambient temperature of 20 °C in order to reduce the simulation time. Thereafter, in the stress relaxation step, the tool was retracted from the workpiece to allow the workpiece material to relax by cooling down to room temperature. As shown in Figure 13, the bottom and left sides of the workpiece are fixed in both horizontal and vertical directions in stress relaxation step. This cooling process was performed using a convection heat transfer to the workpiece consisting of the chip [9,11]. The mechanical and thermal boundary conditions for the cutting and the stress relaxation processes in the FE model for dry and flood-coolant modes as well as the corresponding experimental tests are shown in Figure 12 and Figure 13. The cutting and stress relaxation processes take about 16 and 3 h, respectively. The simulations were carried out using a computer system of Intel^®^ Xeon^®^ CPU E3-1225 V5 with a CPU speed of 3.30 GHz and a memory RAM of 64.0 GB.

## 5. Validation of the FE Model

The developed finite element model was validated by comparing the numerical results of cutting force, thrust force, tool wear, and residual stresses with those obtained through the above-mentioned experimental measurements in dry and flood-coolant modes for Test No. 3 listed in Table 3.

**Figure 14:**
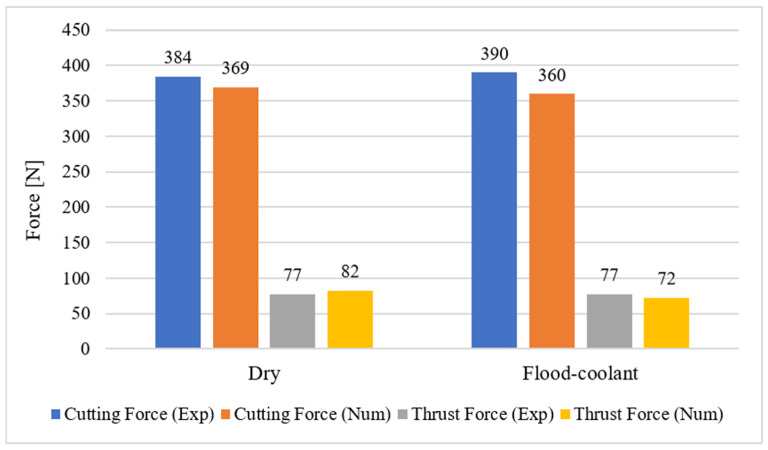
The predicted and experimental cutting forces and thrust forces for dry and flood-coolant cutting.

**Figure 15:**
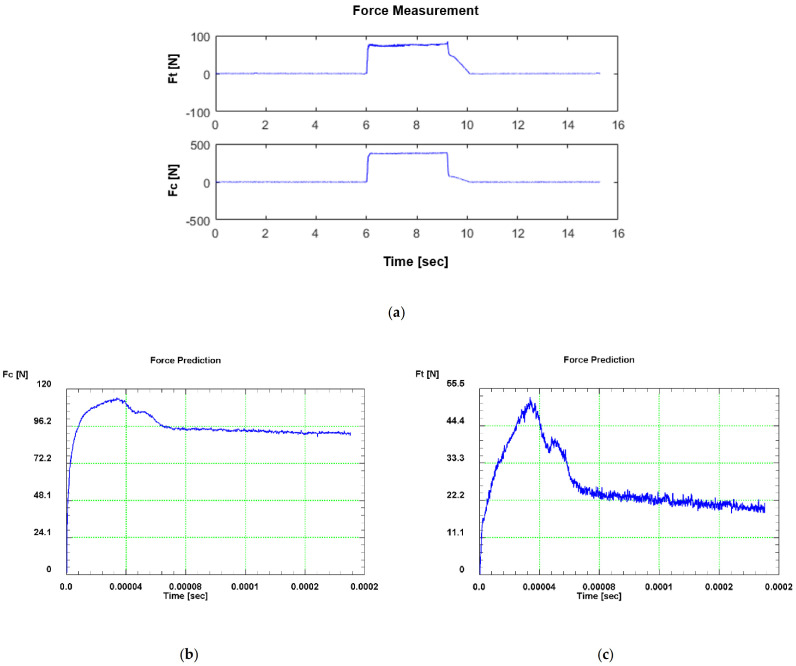
Variation of cutting and thrust forces with time during dry orthogonal cutting: (**a**) in the experiment (force signals) for the 4  mm width of cut and (**b**,**c**) in FE modeling for the unit width of cut.

**Figure 16:**
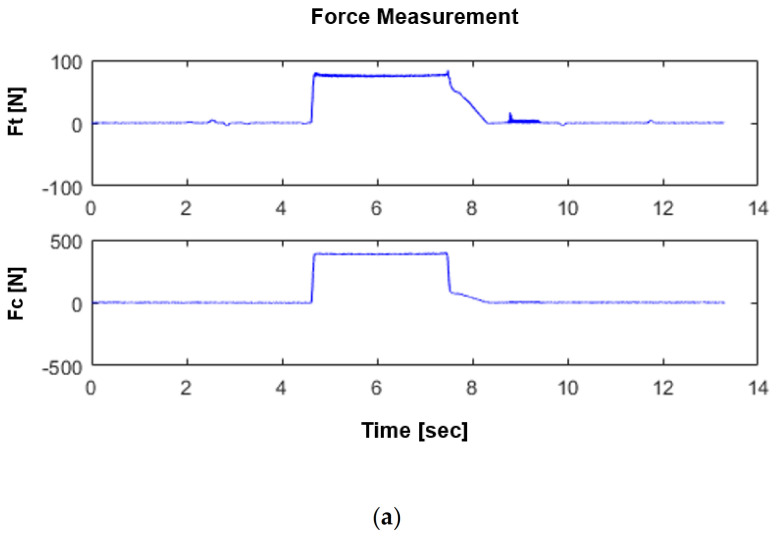

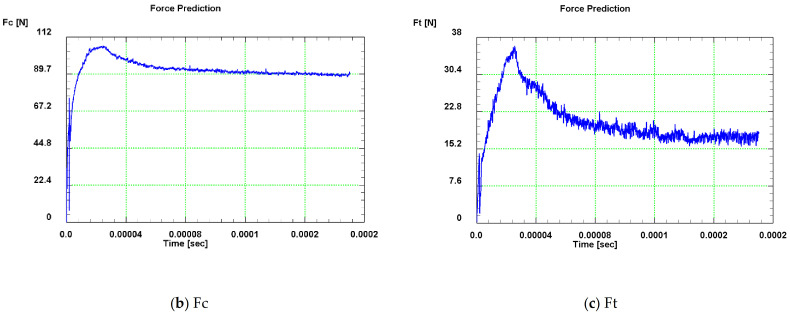
Variation of cutting and thrust forces with time during flood-coolant orthogonal cutting: (**a**) in the experiment (force signals) for the 4 mm width of cut and (**b**,**c**) in FE modeling for the unit width of cut.

The predicted and experimental cutting forces and thrust forces are compared in Figure 14 for both modes. As presented in this figure, there is good agreement between the numerical predictions and experimental results. The variation of the simulated and measured cutting and thrust forces with time are also shown in Figure 15 and Figure 16 in dry and flood-coolant modes, respectively. As observed, the steady-state condition is reached in both simulations and experiments.

**Figure 17:**
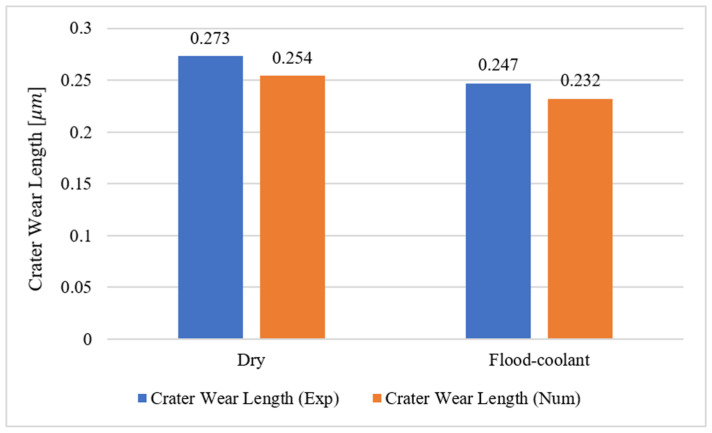
The simulated and measured crater wear for dry and flood-coolant modes.

**Figure 18:**
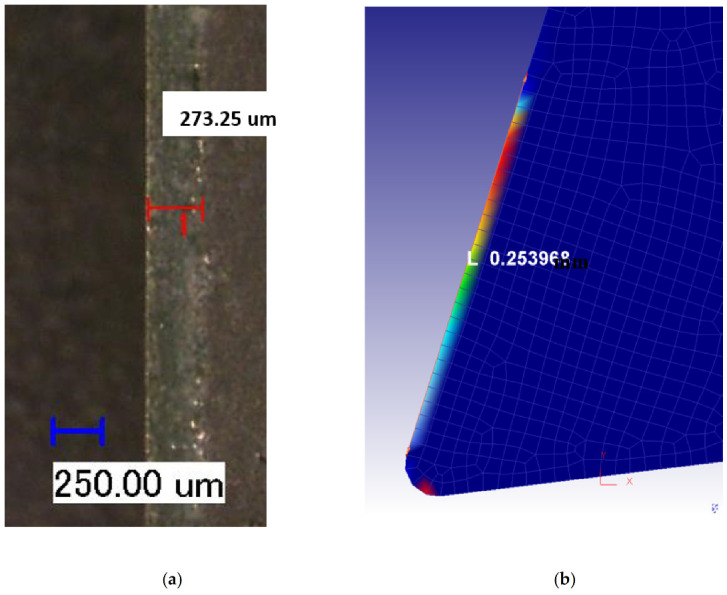
Crater wear for dry mode: (**a**) experimentally observed and (**b**) FE-simulated.

**Figure 19:**
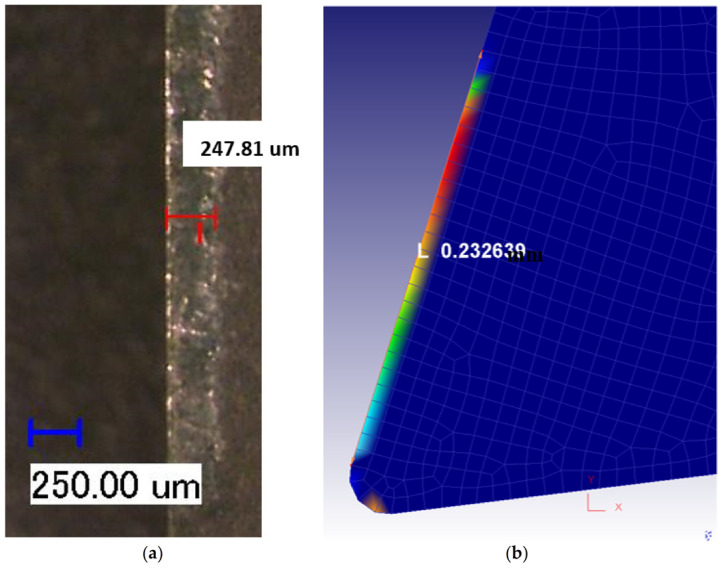
Crater wear for flood-coolant mode: (**a**) experimentally observed and (**b**) FE-simulated.

In addition, as displayed in Figure 17, the simulated and measured crater wear were well matched for both dry and flood-coolant environments. The simulated and measured crater wear are also displayed graphically in Figure 18 and Figure 19 under dry and flood-coolant modes, respectively.

**Figure 20:**
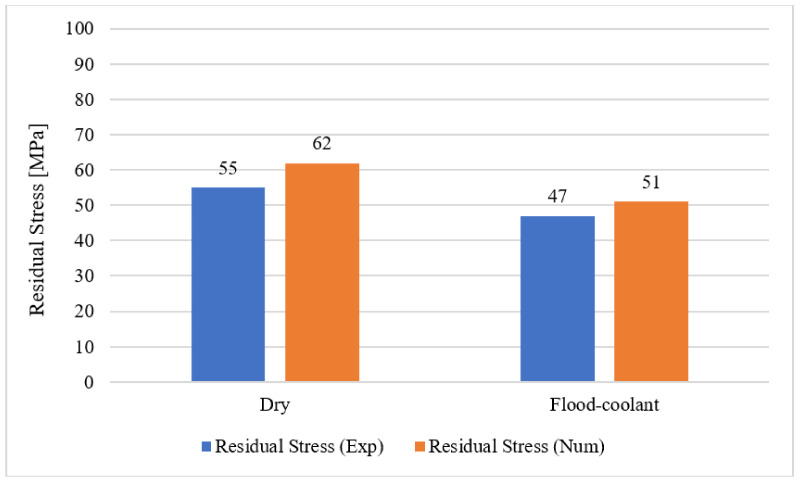
The measured and predicted residual stresses for dry and flood-coolant modes.

**Figure 21:**
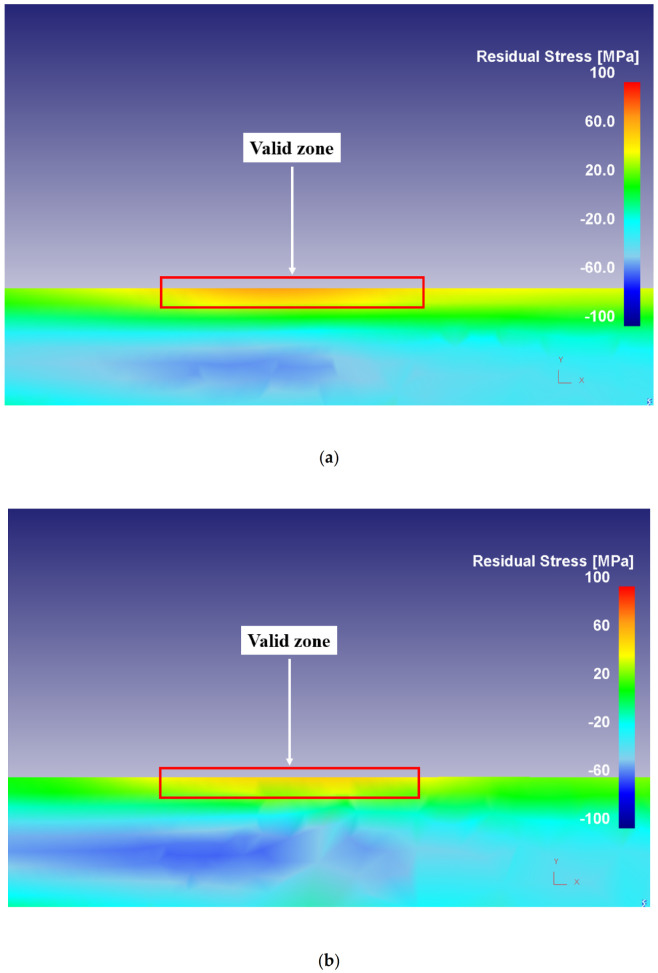
The distribution of simulated residual stress in the machined surface for Test No. 3 in (**a**) dry and (**b**) flood-coolant environments.

As illustrated in Figure 20, good agreement is obtained between the FE results and experimental measurements of residual stresses in dry and flood-coolant modes. The distribution of simulated residual stress in the machined surface for Test No. 3 in dry and flood-coolant environments are shown in Figure 21a,b, respectively.

As observed in the figures related to the validation, the developed FE model was properly validated with the experimental results in dry and flood-coolant modes. This was obtained by exploring various magnitudes of the shear friction factor and heat transfer coefficient and choosing proper coefficients using the calibration of the predicted results with the measured ones, as demonstrated in Table 4. It needs mentioning that in the present study, a heat transfer convection coefficient of 20 $\mathrm{kW}/(m^{2}℃)$ for flood-coolant mode, which was numerically calibrated by [40], was used for FE predictions.

## 6. Summary and Conclusions

The present research studied the impacts of cutting environments and conditions on machining forces, tool wear and residual stresses in the orthogonal cutting of AA6061-T6. Cutting environments consisted of dry and flood-coolant modes and cutting conditions were cutting speed and feed rate. A 2D finite element (FE) model was developed to predict tool wear and residual stresses and was validated with experimentally measured machining forces, tool wear, and residual stresses. The experimental results demonstrated that machining forces almost were not affected by the cutting environment including dry and flood-coolant modes. The experimental results showed that machining forces, tool wear and residual stresses increased with feed rate in both cutting environments. The highest value of tool wear and the lowest value of resultant machining forces and residual stresses were obtained at low speed cutting and high-speed cutting, respectively. Flood-coolant mode improved tool wear, whereas it slightly reduced residual stresses in comparison with dry mode. As a result, cutting with low feed rate and high speed under flood-coolant mode was suggested to improve tool wear and residual stress in the orthogonal cutting of AA6061-T6. The developed 2D finite element model can be used as a predictive tool to simulate tool wear and residual stresses under different cutting environments and conditions to avoid conducting expensive, time-consuming experiment tests and measurements. These results provide the industry with some insights into the cutting conditions and environments to improve the tool performance and the surface quality.

## Figures and Tables

**Table 1:** Cutting conditions for tool geometry including $r_{\beta }$ = 0.02 mm, $\gamma_{o}$ = 17.5°, and $\alpha_{o}$ = 7°.

Test No.	Cutting Speed $V_{C}\left(m/min\right)$	Feed Rate $f\left(mm/rev\right)$
1	361	0.16
2	650	0.16
3	950	0.16
4	1250	0.16
5	950	0.1
6	950	0.2

**Table 2:** The constants of Johnson–Cook material model of AA6061-T6 [9].

$A\;\left(MPa\right)\;$	$B\;\left(MPa\right)\;$	n	C	m	${\dot{\varepsilon }}_{0}\;\left(1/s\right)$	Tmelt (Co)	Troom (Co)
250	79.70	0.499	0.0249	1.499	1	652	20

**Table 3:** Cutting conditions and tool geometry for the validation test.

Test No.	$V_{C}\;\left(m/min\right)$	$f\;\left(mm/rev\right)$	$r_{\beta }\left(mm\right)$	$\gamma_{o}\;\left(deg\right)$	$\alpha_{o}\left(deg\right)$
3	950	0.16	0.02	17.5	7

**Table 4:** The calibrated frictional and thermal coefficients in the FE models.

Coefficient	Dry	Flood-Coolant
Shear Friction Factor	0.98	0.90
Heat Transfer Coefficient $\left(\mathrm{kW}/(m^{2}℃\right))$	10,000	10,000
Heat Convection Coefficient $\left(\mathrm{kW}/(m^{2}℃\right))$	0.02	20

## Data Availability

Data sharing not available.
